# UVB-Induced Secretion of IL-1*β* Promotes Melanogenesis by Upregulating TYR/TRP-1 Expression In Vitro

**DOI:** 10.1155/2022/8230646

**Published:** 2022-05-06

**Authors:** Chun-Yan Yang, Yanni Guo, Wen-Juan Wu, Mao-Qiang Man, Ying Tu, Li He

**Affiliations:** ^1^Department of Dermatology, First Affiliated Hospital of Kunming Medical University, Institute of Dermatology & Venereology of Yunnan Province, Kunming, Yunnan, China; ^2^Baoshan College of Traditional Chinese Medicine, Baoshan, Yunnan, China; ^3^Department of Dermatology, The Second Affiliated Hospital of Fujian Medical University, Quanzhou, China; ^4^Dermatology Hospital, Southern Medical University, Guangdong, China; ^5^Department of Dermatology, University of California San Francisco, San Francisco, California, USA

## Abstract

**Purpose:**

Ultraviolet radiation (UVR) is one of the exogenous stimuli increasing melanogenesis. UV light, especially UVB, is also a potent inducer of epidermal cytokine release. This study is aimed at determining the underlying mechanisms by which UVB-induced cytokines in keratinocytes regulate melanin production *in vitro*.

**Methods:**

Expression levels of mRNA for interleukin- (IL-) 1, IL-1*β*, IL-6, IL-10, IL-17, and tumor necrosis factor-alpha (TNF-*α*) were measured using RT-qPCR at various time points after UVB irradiation in C57BL/6 mice and HaCaT cells. NaOH lysis and L-dihydroxyphenylalanine (L-DOPA) oxidation method were used to measure melanin content and tyrosinase (TYR) activity, respectively, in melanoma B16 cells. RT-qPCR and Western blot were used to assess mRNA and protein levels of microphthalmia-associated transcription factor (MITF), TYR, tyrosine-related protein-1 (TRP-1), and tyrosine-related protein-2 (TRP-2) in B16 cells. Finally, expression levels of cyclooxygenase-2 (COX-2) mRNA and stem cell factor (SCF) in HaCaT cells were measured following knockdown of IL-1*β* using siRNA (siIL-1*β*).

**Results:**

UVB irradiation increased IL-1*β* mRNA expression levels in both C57BL/6 mice and HaCaT cells. The melanin content, TYR activity, and expression levels of TYR and TRP-1 were all raised when B16 cells were treated with 4 pg/l of IL-1. Moreover, IL-1*β* also upregulated the expression levels of SCF and COX-2 in nonirradiated HaCaT cells. Conversely, knockdown of IL-1*β* attenuated UVB irradiation-induced upregulation of SCF and COX-2 expression in keratinocytes.

**Conclusions:**

UVB-induced melanogenesis is mediated in part by IL-1*β*, leading to upregulation of the TYR/TRP1 expression in melanoma B16 cells. IL-1*β* can also stimulate the expression of COX-2 and SCF in HaCaT cells, which in turn increase melanin synthesis in melanocytes. These results suggest that anti-inflammatory approaches could possibly mitigate UVB-induced hyperpigmentation.

## 1. Introduction

Skin consists of the epidermal and dermal layers. The epidermis, the first line of defense protecting the body from external insults, is a multilayered structure consisting of keratinocytes (KCs) that proliferate only in the basal layer and move upward to sequentially form the stratum spinosum, stratum granulosum, and stratum corneum and finally shed off from the top layer of the stratum corneum [1]. Melanocytes (MCs), pigment-producing cells, reside within the basal layer of the epidermis, where they interact through dendritic extensions with more abundant KCs in a ratio of roughly 36 KCs : 1 MC to form the KC : MC pigmentary unit [2, 3]. Melanin, synthesized by MCs, serves as a natural sun screen by absorbing UVR [4]. Among three UV lights, UVC (wavelength 180–280 nm) is completely absorbed by the ozone layer, while both UVA (wavelength 320–400 nm) and UVB (wavelength 280–320 nm) can reach the Earth's surface in sufficient amounts to damage the skin [5]. UVB in particular has a wide spectrum of biological effects on the skin, and its exposure can cause a variety of cutaneous reactions such as hyperpigmentation, erythema, edema, sunburn, hyperplasia, inflammation, and immunosuppression [6]. KCs are the main target of UVB irradiation and play a central role in responses to photodamages, including release of proinflammatory cytokines such as IL-1, IL-6, IL-8, IL-10, and TNF-*α*. It appears that keratinocyte-derived mediators play an important role in the pathogenesis of UV-induced reactions in the skin [7].

Previous studies have shown that the release of secreted factors (cytokines, chemokines, and growth factors) from KCs and other resident skin cells regulates MC proliferation, differentiation, signaling, pigment production and secretion, and dendricity [8]. In epidermal hyperpigmentary disorders, paracrine cytokine interactions between KCs, fibroblasts, and MCs play essential roles in stimulating pigmentation in the epidermis [9]. KCs can influence the melanin production of MCs by producing soluble factors under inflammatory conditions [10]. But the link between inflammation and melanin production upon UV irradiation remains unclear. The cytokines induced by UVB irradiation may directly influence the expression of genes related to melanin synthesis, including MITF, TYR, TRP-1, and TRP-2, in melanocytes. In addition, studies have shown that SCF and COX-2 can also promote melanogenesis [11–13]. In the present study, we determined the contribution of the signaling of keratinocyte origin to UVB-induced pigmentation *in vivo* and *in vitro*.

## 2. Material and Methods

### 2.1. Animals

Female C57BL/6 mice aged 5 to 7 weeks were purchased from Kunming Medical University's animal center (Kunming, China) and fed with mouse diet in our animal facility. Before the experiments, the animals were housed in standardized conditions with a 12-hour light/dark cycle for 1 week. The experimental protocol was approved by the ethical review board of animal welfare of Kunming Medical University, China, and the experiments implemented its guidelines (Laboratory Animal Guideline for ethical review of animal welfare, GB/T 35892-2018).

### 2.2. Cell Culture

HaCaT cells, a human skin keratinocyte line, were purchased from the Cell Bank of Kunming Institute of Zoology, Chinese Academy of Sciences, and cultured in DMEM (11995040, Gibco, USA) supplemented with 10% heat-inactivated fetal bovine serum (FBS) (16140071, Gibco, USA) and antibiotics (100 U/ml penicillin and 100 *μ*g/ml streptomycin; 15140163, Gibco, USA) at 37°C in a humidified incubator containing 5% CO_2_.

B16 cells, a mouse skin melanoma cell line, were provided by the Shanghai Institute of Cell Biology, Chinese Academy of Sciences, and cultured in RPMI 1640 (61870127, Gibco, USA) supplemented with 10% heat-inactivated fetal bovine serum (FBS) (16140071, Gibco, USA) and antibiotics (100 U/ml penicillin and 100 *μ*g/ml streptomycin; 15140163, Gibco, USA) at 37°C in a humidified incubator containing 5% CO_2_.

### 2.3. UVB Irradiation

Back hair of C57BL/6 mice was removed using an electric shaver and Veet hair removal cream one day prior to UVB irradiation. A Solar UV simulator (UV-1000, SIGMA, China) was used to irradiate mice at doses of UVB 120 mJ/cm^2^, and an UVB digital radiometer (SIGMA, China) was used to measure UVB dose. After UVB irradiation, the skin tissue of mice was collected at various time points and stored in RNA later solution (76104, Qiagen, Germany) at -80°C.

HaCaT cells were grown to 70–80% confluence and washed twice with phosphate-buffered saline (PBS) prior to UVB irradiation. The UVBBTL01 lamp (Philips, Amsterdam, Netherlands) was used for UVB irradiation at a dose of 20 mJ/cm^2^. An UVB digital radiometer was used to determine UVB dose. After UVB irradiation, the culture medium was replenished with medium containing 10% heat-inactivated FBS and harvested at various time points.

### 2.4. Real-Time RT-PCR Analysis

Total RNA was prepared using TRIZOL reagent (15596018, Invitrogen, USA), according to the manufacturer's protocol. For the mouse skin, the whole skin (both dermis and epidermis) was sliced into pieces with scissors in TRIZOL reagent (50-100 mg/ml, 15596026, Invitrogen, USA), followed by oscillation with a gyroscopic oscillator for 5 minutes. Samples (1.0 *μ*g RNA) were reverse-transcribed using the FastKing RT kit (KR116, TianGen, China). Synthesized cDNA was subjected to real-time RT-PCR using SYBR Green SuperMix (FP216, TianGen, China). Briefly, after predenaturation for 3 minutes at 95°C, the reaction was cycled for 3 seconds at 95°C and 30 seconds at 60°C. For transcript normalization, beta-actin cDNA served as an internal standard. Each sample was repeated three times and calculated the average cycle threshold (Ct). The real-time fluorescence quantitative was analyzed by the 2^−*ΔΔ*Ct^ method, and data were expressed as folds of control. Primers are listed in Supplemental Table 1.

### 2.5. Western Blotting

After the treatment of B16 cells and HaCaT cells, protein extraction was prepared in RIPA buffer (R0010, Solarbio, China), followed by separation on 10% SDS-PAGE gels. Following conventional protocols, these proteins were then transferred from gel to nitrocellulose membranes. 5% BSA was used to block the membrane at room temperature for 1.5 hours. The membrane was incubated with the respective primary antibody overnight at 4°C. Primary antibodies included anti-tyrosinase (abs131593, Absin, China), anti-TRP-1 (ab235447, Abcam, England), anti-TRP-2 (abs131399, Absin, China), anti-MITF (abs100546, Absin, China), and anti-IL-1*β* (32165, SAB, USA) at a 1 : 1000 dilution. Afterwards, membranes were incubated for 1 hour with 1% skim milk in TBS-T buffer containing horseradish peroxidase-conjugated secondary antibodies (diluted to 1 : 2000). The protein bands were visualized using an ECL system (5200, Tanon, China). The analyzing software (ImageJ) was used to analyze the density of each band. *β*-Actin and *β*-tubulin (ab108342, Abcam, England) were used as control for protein loading.

### 2.6. Cell Proliferation Assay

B16 cells were plated in a 96-well culture plate at a density of 5 × 10^3^ cells/well, in triplicate. Cells were incubated with or without IL-1*β* (0-5 pg/*μ*l) (211-11B-10, PEPROTECH, USA) for 24 hours, followed by addition of 10 *μ*l Cell Counting Kit-8 solution (CCK-8, SAB, USA) to each well for assessment of cell proliferation by measuring absorbance at 450 nm, using a microplate reader.

### 2.7. Quantification of Melanin Content In Vitro

The melanin content of cultured cells was measured as described previously [14]. Briefly, melanoma B16 cells were seeded at a density of 2 × 10^4^ cells/well in 6-well culture plates and incubated at 37°C under 5% CO_2_ atmosphere for 24 hours. The cells were then treated with or without IL-1*β* (2 *μ*g lyophilized was diluted with 2 ml sterile distilled water and diluted with complete medium to the working concentration of 4 pg/*μ*l, 200-01B-2, PEPROTECH, USA) for 48 hours, followed by washing twice with PBS and dissolving in 120 *μ*l of 1 N NaOH (Solarbio, China) containing 10% DMSO (D12345, Gibco, USA) at 80°C for 2 hours. Afterwards, melanin content in homogenates was determined by measuring absorbance at 405 nm using a plate reader (ARVOTM X3; PerkinElmer, Waltham, MA, USA). The amount of cellular melanin was normalized to the number of cells in the samples.

### 2.8. Measurement of Tyrosinase Activity

Tyrosinase activity was measured as described previously [15]. Briefly, B16 cells were seeded at a density of 1 × 10^4^ cells/well in 96-well culture plates and incubated at 37°C under 5% CO_2_ atmosphere overnight. The cells were then treated with or without IL-1*β* (4 pg/*μ*l) for 48 hours, followed by washing twice with PBS and homogenized in 20 mM Tris/HCl (pH 7.5) containing 0.1% Triton X-100 (P1080, Solarbio, China). Tyrosinase activity was assessed by measuring oxidation of L-DOPA to L-dopachrome. Cells were incubated with 100 *μ*l/well of freshly prepared substrate solution (0.1% L-DOPA in PBS) at 37°C for 2 hours. L-dopachrome production was determined by measuring absorbance at 490 nm using a plate reader (ARVOTM X3). The tyrosinase activity was corrected according to the number of cells in the samples.

### 2.9. IL-1*β* Knockdown

HaCaT cells were seeded on 6-well plates at a density of 6 × 10^5^/plate and incubated with IL-1*β* siRNA (customized and designed by GenePharma, China) using Lipofectamine™ 2000 transfection reagent (11668030, Thermo Fisher, USA), according to the manufacturer's instructions. Briefly, cells were incubated with 500 *μ*l/well of freshly prepared solution (10 *μ*l siRNA and 5 *μ*l lipo2000 in DMEM) at 37°C under 5% CO_2_ atmosphere for 24 hours, and then, DMEM supplemented with 10% FBS was added (1 ml/well) and incubated for additional 48 hours. (Negative control: sense 5′-UUCUCCGAACGUGUCACGUTT-3′, antisense 5′-ACGUGACACGUUCGGAGAATT-3′; siIL1*β*-489: forward 5′-GGUGAUGUCUGGUCCAUAUTT-3′, reverse 5′-AUAUGGACCAGACAUCACCTT-3′; siIL1*β*-647: forward 5′-GCGUGUUGAAAGAUGAUAATT-3′, reverse 5′-UUAUCAUCUUUCAACAUGCTT-3′; siIL1*β*-853: forward 5′-GGCCAGGAUAUAACUGACUTT-3′, reverse 5′-AGUCAGUUAUAUCCUGGCCTT-3′.)

### 2.10. Statistical Analysis

Statistical analysis was performed using one-way analysis of variance (ANOVA) or Student's *t*-test. Data are presented as the means ± standard error of the mean (SEM), and the number of samples is stated in figure legends. SPSS one-way analysis of variance 25.0 and GraphPad Prism 7 were used for data analysis.

## 3. Results

### 3.1. UVB Irradiation Increases Expression Levels of Cytokines In Vivo and In Vitro

**Figure 1:**
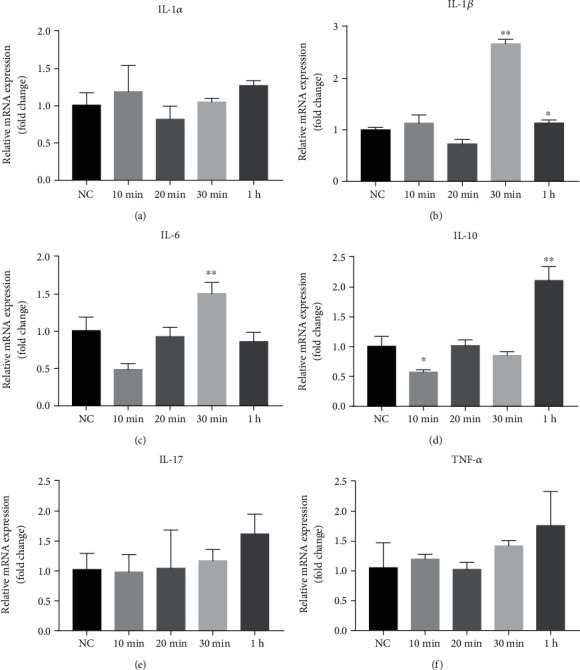
mRNA levels of inflammatory cytokines in C57BL/6 mouse skin at different time points after UVB irradiation at a dose of 120 mJ/cm^2^. The RT-qPCR method was used to detect the mRNA expression of IL-1*α*, IL-1*β*, IL-6, IL-10, IL-17, and TNF-*α* level (a–f). *N* = 3 for all groups. ^∗^*p* < 0.05 and ^∗∗^*p* < 0.01.

Because of the possible role of cytokines in UVR-induced melanogenesis, we first assessed the expression levels of mRNA for cytokines following UVB irradiation in mice. As shown in Figure 1, expression levels of mRNA for IL-1*β*, IL-6, and IL-10 were significantly increased within 1 hour post-UVB irradiation (Figures 1(b)–1(d)), without significant changes in expression levels of IL-1*α*, IL-17, and TNF-*α* (Figures 1(a), 1(e), and 1(f)). Notably, increases in IL-1*β* and IL-6 expression occurred earlier than IL-10, suggesting IL-1*β* and IL-6 could be the upstream signaling molecules stimulating melanogenesis following UVB irradiation.

**Figure 2:**
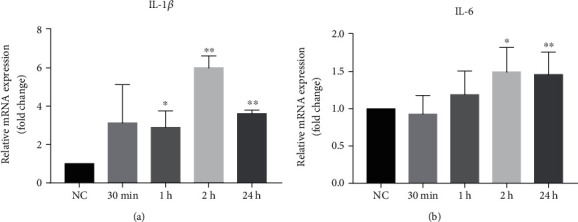
Expression of IL-1*β* and IL-6 mRNA in HaCaT cells at different time points after UVB irradiation with UVB 20 mJ/cm^2^. Expression levels of IL-1*β* and IL-6 mRNA were detected by the RT-qPCR method at 30 minutes, 1 hour, 2 hours, and 24 hours, respectively. (a) Expression levels of IL-1*β*. (b) Expression levels of IL-6. *N* = 6 for all groups. ^∗^*p* < 0.05 and ^∗∗^*p* < 0.01.

Because keratinocytes are the main target cells for UVB radiation, next we assessed expression levels of IL-1*β* and IL-6 mRNA in HaCaT cells following UVB irradiation. Compared to the controls, a significant increase in IL-1*β* expression began 1 hour after irradiation (Figure 2(a)), while a significant elevation in IL-6 mRNA appeared 2 hours after UVB irradiation (Figure 2(b)). UVB-induced increases in both IL-1*β* and IL-6 mRNA expression sustained at least 24 hours.

### 3.2. IL-1*β* Increases Melanin Content in B16 Cells

**Figure 3:**
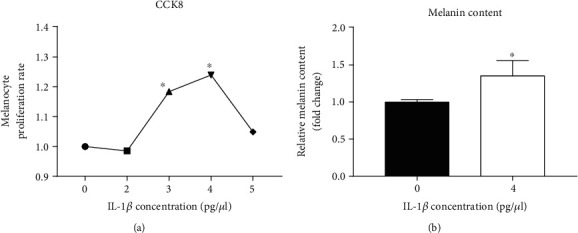
Effects of IL-1*β* on B16 cell proliferation and melanin content. (a) Proliferation rates of B16 cells following the treatment with different concentrations of IL-1*β* (2 pg/*μ*l, 3 pg/*μ*l, 4 pg/*μ*l, and 5 pg/*μ*l) for 48 hours. (b) The melanin content of B16 cells was measured by the NaOH method after treatment with 4 pg/*μ*l IL-1*β* medium for 48 hours. *N* = 3 for all groups. ^∗^*p* < 0.05.

As shown below, UVB radiation-induced IL-1*β* expression increase appeared early both *in vivo* and *in vitro*, suggesting that IL-1*β* is likely the upstream signaling in UVB-induced melanogenesis. To test this hypothesis, melanin content was measured in B16 cells after the treatment with IL-1*β*. Because the proliferation rate of B16 cells was higher at IL-1*β* concentration of 4 pg/*μ*l (Figure 3(a)), melanin content was measured in B16 cells cultured with 4 pg/*μ*l of IL-1*β* for 48 hours. As expected, treatment of B16 cells with IL-1*β* increased melanin content by 46% over the vehicle controls (Figure 3(b), *p* < 0.05).

**Figure 4:**
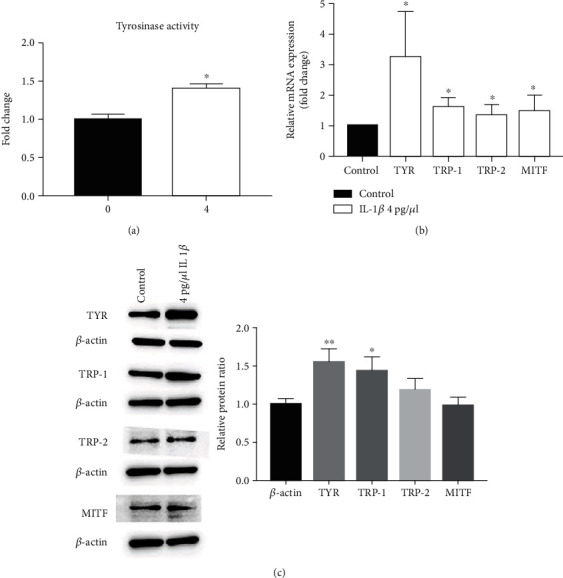
Effect of IL-1*β* on tyrosinase activity and expression levels of MITF, TYR, TRP-1, and TRP-2 mRNA and protein in B16 cells after treatment with 4 pg/*μ*l IL-1*β* for 48 hours. (a) Tyrosinase activity. (b) Expression levels of MITF, TYR, TRP-1, and TRP-2 mRNA. (c) Expression levels of MITF, TYR, TRP-1, and TRP-2 protein. *N* = 3 for all groups. ^∗^*p* < 0.05 and ^∗∗^*p* < 0.01.

To elucidate the potential mechanisms by which IL-1*β* increases melanin production, we determined next whether IL-1*β* increases expression and activity of key enzymes for melanin synthesis, including MITF and tyrosinase family. Indeed, incubation of B16 cells with IL-1*β* markedly increased tyrosinase activity (Figure 4(a)) and expression levels of mRNA for MITF, TYR, TRP-1, and TRP-2 (Figure 4(b)). In parallel, expression levels of TYR and TRP-1 protein were also significantly increased (Figure 4(c)). These results demonstrate that IL-1*β* increases expression and activity of key enzymes for melanin synthesis.

### 3.3. IL-1*β* Upregulates COX-2 and SCF mRNA Expression in HaCaT Cells

**Figure 5:**
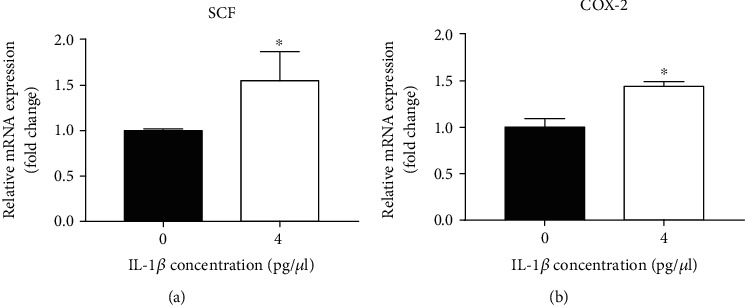
Expression levels of SCF and COX-2 mRNA after IL-1*β* treatment. After HaCaT cells were cultured with 4 pg/*μ*l IL-1*β* for 24 hours, RT-qPCR was used to measure expression levels of SCF and COX-2 mRNA. (a) Expression levels of SCF. (b) Expression levels of COX-2. *N* = 3 for all groups. ^∗^*p* < 0.05.

Because both SCF and COX-2 can stimulate melanogenesis [12, 13], next we determined whether IL-1*β* increases SCF and COX-2 in HaCaT cells. Our results showed that the expression levels of SCF and COX-2 mRNA significantly increased after incubation with 4 pg/*μ*l IL-1*β* (*p* < 0.05 vs. controls) (Figure 5).

**Figure 6:**
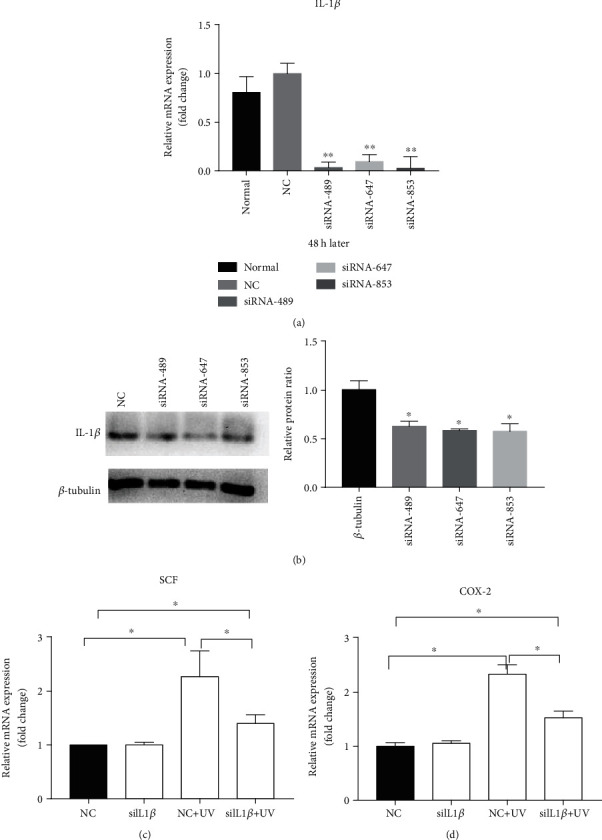
Effect of IL-1*β* knockdown on the expression of SCF and COX-2 in HaCaT cells. (a, b) The effects of siRNA489 on expression levels of IL-1*β* mRNA and protein, respectively. (c, d) The inhibitory effect of siRNA489 on UVB-induced increases in SCF and COX-2 expression in HaCaT cells. *N* = 3 for all groups. ^∗^*p* < 0.05 and ^∗∗^*p* < 0.01.

To determine the role of IL-1*β* in UVB-induced increases in SCF and COX-2, we employed siRNA to knock down IL-1*β* in HaCaT cells. As seen in Figures 6(a) and 6(b), IL-1*β* siRNA decreased expression levels of both mRNA and protein in keratinocytes (Figures 6(a) and 6(b)). While UVB irradiation increased SCF and COX-2 mRNA expression, IL-1*β* siRNA attenuated the effects of UVB on SCF and COX-2 expression (Figures 6(c) and 6(d)). These results suggest that UVB-induced increases in SCF and COX-2 are mediated, at least in part, by IL-1*β* secreted by keratinocytes, which in turn regulates melanogenesis in melanocytes.

## 4. Discussion

It is well known that UVR can induce cutaneous inflammation. Previous studies have shown that UVR (especially UVB) exposure stimulates keratinocytes to secrete abundant proinflammatory cytokines, including IL-1, IL-6, IL-10, and TNF-*α* [16], which can induce a secondary cascade of mediators and cytokines from keratinocytes and other cells resulting in wide range of innate processes such as infiltration of inflammatory leukocytes, induction of immunosuppression, DNA repair, or apoptosis [17]. UVR-induced increases in TNF-*α* and IL-17 are key mediators of UVR-induced cutaneous and systemic inflammation [18, 19]. IL-1 can increase release of other cytokines such as IL-6 by autocrine or paracrine in keratinocytes after ultraviolet irradiation [20]. Correspondingly, the present study showed that UVR increased IL-1*β* as early as 30 minutes while increasing IL-10 in mice one hour after UVB irradiation. In contrast, IL-1*α* remained no significant changes within one hour after UVB irradiation. Thus, IL-1*β* is likely upstream signaling in the cascade of UVB-induced cutaneous inflammation.

In addition to inflammation, UVR can also stimulate melanogenesis. But the underlying mechanisms by which UVR induces skin pigmentation are unclear. Melanin synthesis comprises a series of complex processes regulated by various factors, including TYR, MITF, TRP-1, and TRP-2 [4]. IL-1*β* can promote ultraviolet-induced pigmentation by promoting the secretion of *α*-MSH and ET-1 [21]. The present study showed that IL-1*β* not only increased the expression levels of MITF, TRP-1, and TRP-2 but also increased the activity of TYR and melanin content in B16 cells, in addition to stimulation of B16 cell proliferation. Thus, UVB-induced melanogenesis can be ascribed to the direct effects of IL-1*β* on expression of protein-related to melanin production and stimulation of melanocyte proliferation. Moreover, while SCF and COX-2, mediators that promote melanogenesis, can be regulated by IL-1*β* in other cell lines [22, 23], we observed the effect of IL-1*β* on the expression of SCF and COX-2 in HaCaT cells. Both UVR and IL-1*β* increased expression levels of SCF and COX-2 in keratinocytes, consistent with previous reports [24, 25]. It is worth noting that knockdown of IL-1*β* largely prevented UVR-induced increase in SCF and COX-2 expression, suggesting role of IL-1*β* in mediating inductions of SCF and COX-2 expression by UVR. While binding of SCF to its receptor c-KIT plays an important role in melanogenesis [26, 27], COX-2 also mediates UVB-induced melanin production [28]. Hence, UVR-induced increase in IL-1*β* can indirectly stimulate melanogenesis.

UVB-induced inflammation and postinflammatory hyperpigmentation (PIH) are complex processes involving both molecular and cellular changes that lead to overproduction of melanin [29]. As we all know, excessive skin pigmentation after inflammation is a common clinical symptom and challenging to treat. Melanin synthesis inhibitors, including kojic acid and its derivatives (such as kojic acid ether derivatives), are TYR inhibitors. However, these agents only target the downstream signaling in melanin production pathway. In addition, these agents have serious side effects such as weak carcinogenicity and discoloration. Finding new and safe melanin production inhibitors has potential applications in medicine and cosmetics [30]. The results of the present study suggest that IL-1*β* is likely the upstream molecule that regulates the UVB-induced hyperpigmentation, by increasing the expression levels of melanogenesis-related genes and possibly other inflammatory factors.

## 5. Conclusion

UVR increases IL-1*β* expression in both the epidermis of C57BL/6 mice and HaCaT cells, which can directly stimulate melanogenesis by increasing the expression levels of melanogenesis-related genes in B16 cells and indirectly stimulate melanogenesis by upregulation of the expression of SCF and COX-2 in HaCaT cells. However, whether anti-inflammation can alleviate or prevent UVR-induced hyperpigmentation remains to be determined in the clinical setting.

## Data Availability

Readers can access the data supporting the conclusions of the study by contacting the author (email: yangchunyan2018@126.com).

## Supplementary Materials

Supplementary Materials Supplemental Table 1: primers used for RT-qPCR.
