# Supplementary material for: UVB-Induced Secretion of IL-1β Promotes Melanogenesis by Upregulating TYR/TRP-1 Expression In Vitro
Source: Biomed Res Int. 2022 May 6;2022:8230646. doi: 10.1155/2022/8230646 (PMC9106468; doi:10.1155/2022/8230646)
Supplement: Supplementary Materials — Supplemental Table 1: primers used for RT-qPCR. [file 8230646.f1.docx]

**Supplemental Table 1 Primers used for RT-qPCR**

| Species | Gene names | Forward primer (5’- 3’) Reverse primer (5’- 3’) |
| --- | --- | --- |
| Mouse | *β-Actin* | TATGGAATCCTGTGGCATC GTGTTGGCATAGAGGTCTT |
| Mouse | *Tnf-α* | TTCTGTCTACTGAACTTC CCATAGAACTGATGAGAG |
| Mouse | *Il-1α* | GTGTTGCTGAAGGAGTTG ATGTGAAGTAGTTCTTAGAGTTG |
| Mouse | *Il-1β* | CAATGGACAGAATATCAAC ACAGGACAGGTATAGATT |
| Mouse | *Il-6* | ACCTGTCTATACCACTTC GCATCATCGTTGTTCATA |
| Mouse | *Il-10* | AGCAGGTGAAGAGTGATT GCAGTTGATGAAGATGTCA |
| Mouse | *Il-17* | TTCTGTCTACTGAACTTC CCATAGAACTGATGAGAG |
| Mouse | *Tyr* | GAAGCGAGTCTTGATTAG AGGTCGTAGATGTTGATA |
| Mouse | *Mitf* | GTATGAACACGCACTCTC TTGATTCCAGGCTGATGA |
| Mouse | *Trp-1* | GCTGCGTTGTTACTTGTA CTTCATTCTTGGTGCTTCT |
| Mouse | *Trp-2* | TACAATTACGCCGTTGAT GATTCCAATGACCACTGA |
| Human | *β-Actin* | CACCATTGGCAATGAGCGGTTC AGGTCTTTGCGGATGTCCACGT |
| Human | *Il-1β* | CCACAGACCTTCCAGGAGAATG GTGCAGTTCAGTGATCGTACAGG |
| Human | *Scf* | CTGGAGACTCCAGCCTACACTG CTGCCCTTGTAAGACTTGGCTG |
| Human | *Cox-2* | CGGTGAAACTCTGGCTAGACAG GCAAACCGTAGATGCTCAGGGA |
